# 1181. Serotype Distribution by Age of Remaining Invasive Pneumococcal Disease After Long-Term PCV10/13 Use: The PSERENADE Project

**DOI:** 10.1093/ofid/ofab466.1374

**Published:** 2021-12-04

**Authors:** Maria Garcia Quesada, Marissa Hetrich, Maria Deloria Knoll

**Affiliations:** Johns Hopkins Bloomberg School of Public Health, Baltimore, Maryland

## Abstract

**Background:**

Pneumococcal conjugate vaccines (PCV) have reduced invasive pneumococcal disease (IPD) (see other PSERENADE abstract), of which > 70% was vaccine-type pre-PCV. We described the serotype (ST) distribution of remaining IPD in countries with mature infant PCV10/13 programs.

**Methods:**

IPD ST distribution data were obtained directly from surveillance sites, supplemented with published literature. Mature programs were defined as exclusive use of PCV10 or PCV13 for at least 5-7 years (depended on if prior PCV7 use and/or PCV10/13 catch-up) with primary series uptake > 70%. The distribution was estimated using a multinomial Dirichlet regression, stratified by PCV product and age (< 5 years, ≥ 50 years).

**Results:**

Serotyped IPD cases from 42 PCV13- (n=78,912) and 12 PCV10-using sites (n=8,429) in 41 countries were analyzed. Most sites were from high-income countries (67%) and used a booster dose schedule (81%). For low- and middle-income countries, only 5 and 7 sites had more than 20 eligible cases for children and adults, respectively. In PCV10 sites, 10.0% (95% CI: 6.3-12.9%) and 15.5% (95% CI: 13.4-19.3%) of the remaining IPD during the mature period was PCV10-type among children and adults, respectively (Figure 1). For PCV13 sites, PCV13-type was 26.4% (95% CI: 21.3-30.0%) among children and 29.5% (95% CI: 27.5-33.0%) among adults. PCV20-, PCV24-, and PPV23-type cases ranged from 62-72% across all age and PCV-use groups. ST 19A was the leading ST at PCV10 sites, though more so for children (30.6%, 95% CI: 18.2-43.1%) than adults (14.8%, 95% CI: 11.9-17.8%; Figure 2). ST 3 was a top ST in both PCV10 and PCV13 sites, causing about 9% of cases in children and 14% in adults. ST 6C was the third most common ST in PCV10 sites, causing 6% of cases in both age groups. Some top non-PCV13 STs are included in higher-valent investigational PCVs (15BC, 12F, 22F, 8, 9N) but others are not (24F, 23B, 23A, 15A).

Figure 1. Percentage of IPD cases in the mature PCV10/13 period due to serotypes included in current and upcoming products.

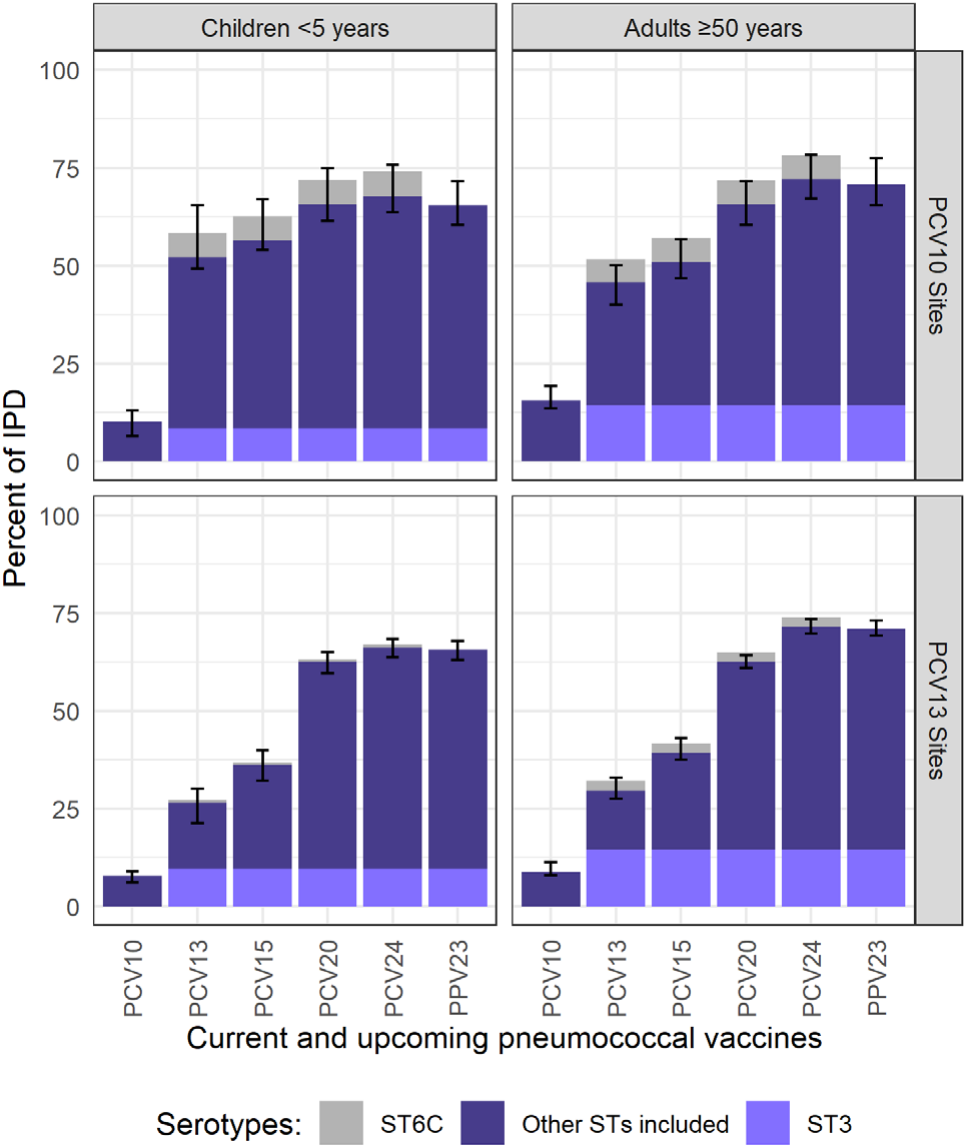

Serotype (ST) 3 is illustrated separately in lighter purple in the bars corresponding to products that include ST3 due to the uncertain effectiveness against ST3 in current products. ST6C is illustrated in grey above the bars where ST6A is included. Although ST6C is not included in PCV10 or PCV13, PCV13 offers cross-protection through ST6A. ST6A also benefits from cross-protection with ST6B, included in both PCV10 and PCV13. Therefore, ST6A causes a very small fraction of disease in both settings and age groups, and it is not shown. Confidence intervals do not include ST6C, as this serotype is not included in PCV10/13. PCV13 is Pfizer’s Prevnar13/Prevenar13; PCV10 is GSK’s Synflorix.

Figure 2. Serotype-specific distribution of IPD in the mature PCV10/13 period.

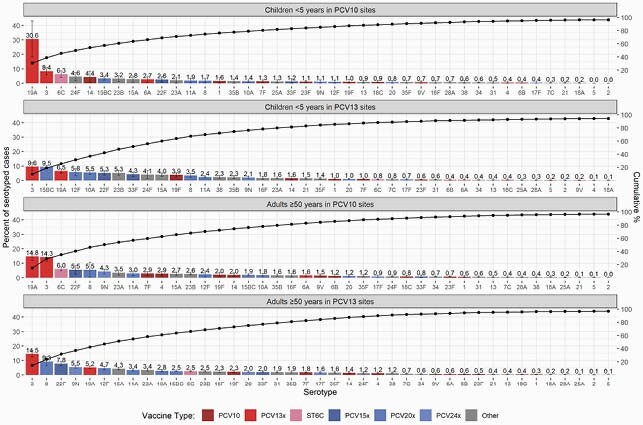

Serotypes are colored by the lowest valency PCV product they are included in. The “x” in the PCV legend represents the extra serotypes included in that product relative to the next lower product (i.e., PCV13x includes serotypes 3, 6A, and 19A not in PCV10). Serotype (ST) 6C is colored separately because, although it is not included in any product, it is covered through cross-protection with PCV13-type serotype 6A. PCV13 is Pfizer’s Prevnar13/Prevenar13; PCV10 is GSK’s Synflorix.

**Conclusion:**

IPD due to vaccine STs was low for both children and adults in countries with mature PCV programs. ST distribution of remaining IPD differed between PCV10 and PCV13 sites and between age groups. Higher-valency PCVs under evaluation target over half of remaining IPD cases, but some prevalent STs are not included in known investigational products.

**Disclosures:**

**Maria Deloria Knoll, PhD**, **Merck** (Research Grant or Support)**Pfizer** (Research Grant or Support)

